# Cancer-Associated Fibroblast Subtypes Reveal Distinct Gene Signatures in the Tumor Immune Microenvironment of Vestibular Schwannoma

**DOI:** 10.3390/cells13191669

**Published:** 2024-10-09

**Authors:** Ji-Yong Sung, Jung Woo Lee

**Affiliations:** 1 Department of Research & Development, VeraOmics, Seoksanro 138, Namdong-Gu, Incheon 21551, Republic of Korea; 2 Department of Orthopedic Surgery, Wonju College of Medicine, Yonsei University, Wonju 26426, Republic of Korea; 3 Yonsei Institute of Sports Science and Exercise Medicine, Wonju 26426, Republic of Korea

**Keywords:** cancer-associated fibroblasts, CAF subtypes, metabolic reprogramming, stem-like CAF, vestibular schwannoma

## Abstract

Cancer-associated fibroblast (CAF) composition within the same organ varies across different cancer subtypes. Distinct CAF subtypes exhibit unique features due to interactions with immune cells and the tumor microenvironment. However, data on CAF subtypes in individuals with vestibular schwannoma (VS) are lacking. Therefore, we aimed to distinguish CAF subtypes at the single-cell level, investigate how stem-like CAF characteristics influence the tumor immune microenvironment, and identify CAF subtype-specific metabolic reprogramming pathways that contribute to tumor development. Data were analyzed from three patients with VS, encompassing 33,081 single cells, one bulk transcriptome cohort, and The Cancer Genome Atlas Pan-Cancer database (RNA sequencing and clinical data). Our findings revealed that antigen-presenting CAFs are linked to substantially heightened immune activity, supported by metabolic reprogramming, which differs from tumorigenesis. High expression of the stem-like CAF gene signature correlated with poor prognosis in low-grade gliomas within the pan-cancer database. This is the first study to classify CAF subtypes in VS patients and identify a therapeutic vulnerability biomarker by developing a stem-like CAF gene signature. Personalized treatments tailored to individual patients show promise in advancing precision medicine.

## 1. Introduction

Vestibular schwannoma is the most common cerebellopontine angle benign tumor and originates from the cochleao-vestibular nerve. Although VS is benign, when located in a fatal area, it can induce cranial complications such as hearing loss, facial palsy, hydrocephalus, and possible mortality. So, the most important prognostic factor associated with VS is tumor growth. Many researchers have tried to find a prediction marker for tumor growth, but no established markers exist yet.

Cancer-associated fibroblasts (CAFs) play a pivotal role in the tumor microenvironment and influence tumor growth and progression. They are the most common stromal cells, and their presence is linked to poor prognosis [1]. Malignant transformation is often preceded by an initial noticeable growth of fibroblasts, which is typically observed in early or precancerous tumors [2]. The progressive nature of these transformations led to the hypothesis that most stromal fibroblasts originally arise from localized fibroblasts subjected to tissue dysfunction [3].

CAFs exhibit a wide range of characteristics, origins, and functions and can be further subdivided into several subgroups, with the primary subgroups being myofibroblastic CAFs (myoCAFs), immune regulatory/inflammatory CAFs (infCAFs), and antigen-presenting CAFs (apCAFs). The heterogeneity of these subgroups has been extensively studied using single-cell RNA sequencing (RNA-seq) technologies [4].

The extracellular matrix (ECM) is a complex structure composed of extracellular molecules that form a tissue-supportive physiological matrix and influence the architecture and function of the extracellular space in malignant tumors and distant metastases. It is a critical factor contributing to the formation of the tumor microenvironment (TME). CAFs primarily mediate the accumulation, modification, and degradation of the ECM. Pathological changes linked to poor prognosis in patients with breast, pancreatic, and lung cancers are attributed to dysregulated ECM remodeling by CAFs [5]. CAFs are related to myofibroblasts and acquire unique muscle contraction functions (characterized by high expression of smooth muscle actin) during wound healing [6,7]. Fibroblasts can detect physical stress and cellular damage signals, subsequently promoting cell regeneration and repair through ECM biosynthetic pathways, while also regulating immune responses.

By modulating the tumor immune microenvironment (TIME), CAFs promote tumor progression, immune evasion, and malignant transformation by suppressing cytokines and chemokines, recruiting and retaining inhibitory myeloid and regulatory T cells, inhibiting and excluding cytotoxic leukocytes and dendritic cells, and facilitating the polarization of macrophages and T cells into their anti-inflammatory M2 and Th2 phenotypes. Moreover, the Warburg effect, characterized by increased glucose uptake and lactic acid production and decreased oxygen consumption, has been observed in CAFs. CAFs respond to glutamine deficiency, and their metabolic heterogeneity has been thoroughly investigated [8]. We previously reported that various CAF subtypes employ different metabolic reprogramming mechanisms, along with their mechanistic associations with tumor development [9].

Currently, there is limited information on whether the immunoregulatory functions of CAFs, such as inducing inflammation and immunosuppression, are specific to certain subgroups or if universal features are shared across cancer types and organs. Furthermore, studies on CAF subtypes in vestibular schwannoma (VS) remain limited. Therefore, we aimed to classify patients with VS based on their CAF features to identify differences in the TIME. Additionally, this study sought to establish a foundation for patient classification by CAF subtypes, facilitating precision medicine and the development of therapeutic strategies for VS.

## 2. Materials and Methods

### 2.1. Single-Cell Analysis

For the single-cell analysis, 2373 fibroblasts were extracted from 33,081 cells derived from three patients [10] using the R package “Seurat” [11]. Single-cell RNA sequencing (RNA-seq) data from a previous study [10] were provided by Dr. Feng Liu (liufeng@sibs.ac.cn). CAF cell types were classified with the default settings of the uniform manifold approximation and projection (UMAP) algorithm in the “Seurat” R package, based on the well-established CAF marker genes across all cell types [10]. Roswell Park Memorial Institute 1640 medium was used and transferred to an enzymatic hydrolysate for tumor hydrolase. HFDM-1 (human fibroblast culture medium) was used for fibroblast dissociation, and alpha smooth muscle actin, actin alpha 2, and vimentin served as markers for CAF identification. In this study, adult patients with sporadic unilateral medium-sized tumors were enrolled. 

Gene ontology analysis for marker genes in each cluster was conducted using the Metascape platform [12], applying the default values of the “Seurat” package, and protein–protein interaction (PPI) networks were identified with the MCODE algorithm. Pathway gene set activity was analyzed using the R package “GSVA” [13], with *p*-values computed from one million simulations of altered expression profiles to assess the statistical significance of the scores. RaceID and StemID [14] were employed to evaluate fibroblast stemness.

### 2.2. Bulk Gene Expression Data Analysis

The VS cohort from the Gene Expression Omnibus repository (GSE141801) was used to perform bulk gene expression analysis, and immune scores were calculated via deconvolution analysis using xCell [15]. Classification into high- or low-immunity groups was determined based on the average value: samples with values higher than the average were placed in the high-immunity group, while those with lower values were classified into low-immunity group. Kyoto Encyclopedia of Genes and Genomes (KEGG) pathway enrichment analysis was performed to assess group-specific pathway activity. Fifty hallmark cancer-related pathways from MSigDB were used for enrichment analysis. Furthermore, the web-based GEPIA2 platform (http://gepia2.cancer-pku.cn/#index, accessed on 1 August 2023) [16] was utilized to analyze gene signature expression and predict prognosis using The Cancer Genome Atlas (TCGA) Pan-Cancer data. Genes with a *p*-value < 0.01 and a fold change ≥ 1 were considered differentially expressed.

## 3. Results

### 3.1. CAF Subtypes Reveal Different Gene Signatures at the Single-Cell Level

**Figure 1:**
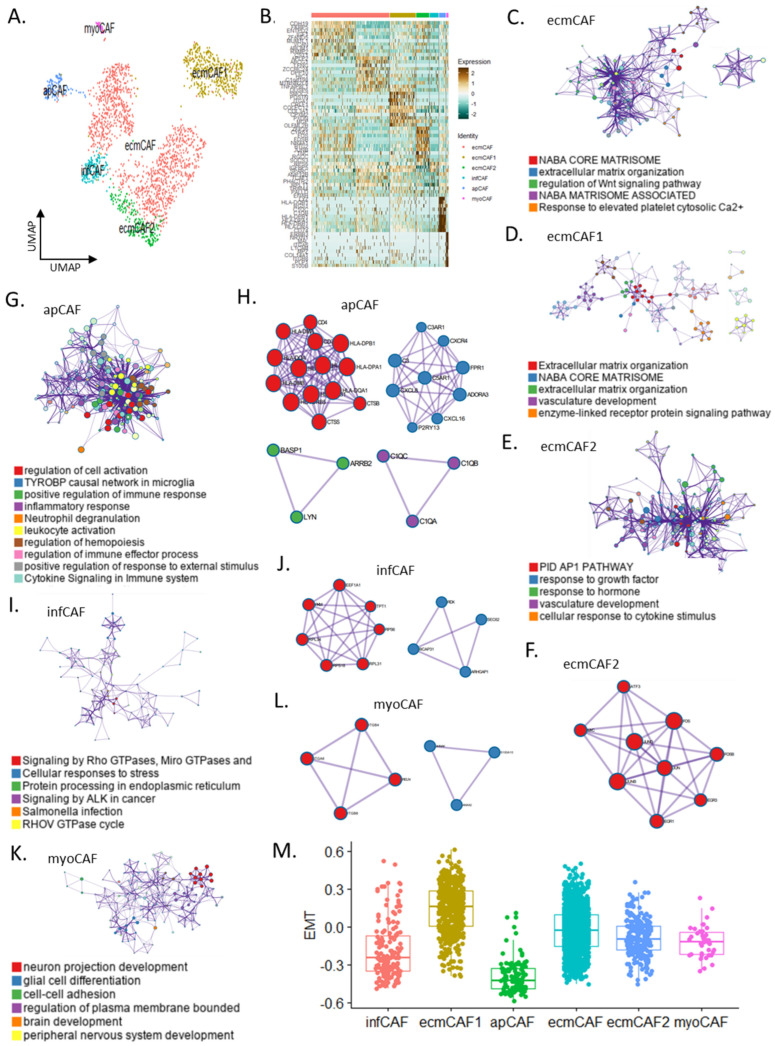
CAF subtypes reveal distinct gene signatures at the single-cell level. (**A**) Uniform manifold approximation and projection of CAF subtypes for VS. (**B**) Heat map of CAF subtypes (ecmCAF, ecmCAF1, ecmCAF2, infCAF, apCAF, and myoCAF). (**C**–**E**) Gene ontology networks for ecmCAF (**C**), ecmCAF1 (**D**), and ecmCAF2 (**E**). (**F**,**G**) PPI (**H**) and gene ontology (**G**) networks for ecmCAF2. (**I**,**J**) Gene ontology (**I**) and PPI (**J**) networks for infCAF. (**K**,**L**) Gene ontology (**K**) and PPI (**L**) networks for myoCAF. (**M**) Box plot for EMC score in CAF subtypes. CAF, cancer-associated fibroblast; VS, vestibular schwannoma; ecmCAF, extracellular matrix CAF; infCAF, immune regulatory/inflammatory CAF; apCAF, antigen-presenting CAF; myoCAF, myofibroblastic CAF; PPI, protein–protein interaction.

Using single-cell data from 2373 fibroblasts, we identified six CAF subgroups: ecmCAFs, ecmCAF1, ecmCAF2, infCAFs, apCAFs, and myoCAFs (Figure 1A). Based on their heterogeneity, we further classified ECM CAFs into ecmCAFs, ecmCAF1, and ecmCAF2. The top 10 marker genes for each subtype were selected [ecmCAF (*CDH19*, *FKBP5*, and *ENTPD2*), ecmCAF1 (*SMOC2*, *POSTN*, and *ITGA1*), ecmCAF2 (*ZFP36*, *CYR61*, and *FOS*), infCAF (*RPS6*, *IGFBP5*, and *SPARC*), apCAF (*LYZ*, *HLA-DQB1*, *HLA-DPB1*, *HLA-DPA1*, and *CD74*), and myoCAF (*ERBB3*, *NRXN1*, and *COL14A1*)] (Figure 1B). All ECM CAFs were connected to the NABAcore matrisome (Figure 1C,D); however, the gene profile of ecmCAF2 was enriched for activator protein 1 (AP1), vascular development biological pathways, and responses to growth factors and hormones (Figure 1E). The gene profiles of ECM CAF subtypes were enriched in Wnt signaling pathway regulation, response to the elevated platelet cytosolic Ca^2+^ pathway, and enzyme-linked receptor protein signaling system activity. The PPI of ecmCAF2 was related to the AP1 pathway, nerve growth factor-stimulated transcription, and the transforming growth factor-beta signaling pathway (Figure 1F). Notably, apCAFs had distinct characteristics. The antigen-presenting CAF signatures were enriched for cell activation regulation, positive immune response regulation, the inflammatory response, neutrophil degranulation, leukocyte activation, and cytokine signaling in the immune system (Figure 1G). According to the PPI analysis, apCAFs were mainly classified into four categories related to antigen processing and presentation, G alpha signaling events, the C1q complex, and synapse pruning (Figure 1H). ApCAFs are primarily regulated by the transcription factors RFXANK, RFXAP, and RFX5 (Supplementary Figure S1A). The InfCAF signatures were mostly enriched in Rho GTPase signaling, anaplastic lymphoma kinase signaling in cancer, and RHOV GTPase cycling, whereas their PPI phenotypes were primarily linked to peptide chain elongation and cellular responses to stress (Figure 1I,J). PPI analysis of the myoCAFs revealed that they were linked to ECM–receptor interactions and positive regulation of the plasma membrane and that they were enriched in neuron projection development, glial cell differentiation, and cell–cell adhesion (Figure 1K,L). MyoCAFs are primarily regulated by POU5F1, TCF4M, and TFAP2A (Supplementary Figure S1B). Epithelial–mesenchymal transition genes were enriched in ecmCAF1 (Figure 1M). Our findings are the first to establish the molecular signature of CAF subtypes at the single-cell level and identify the pathways involved in tumorigenesis.

### 3.2. CAF Subtypes Exhibit Varied Metabolic Reprogramming in Different Immune Statuses

**Figure 2:**
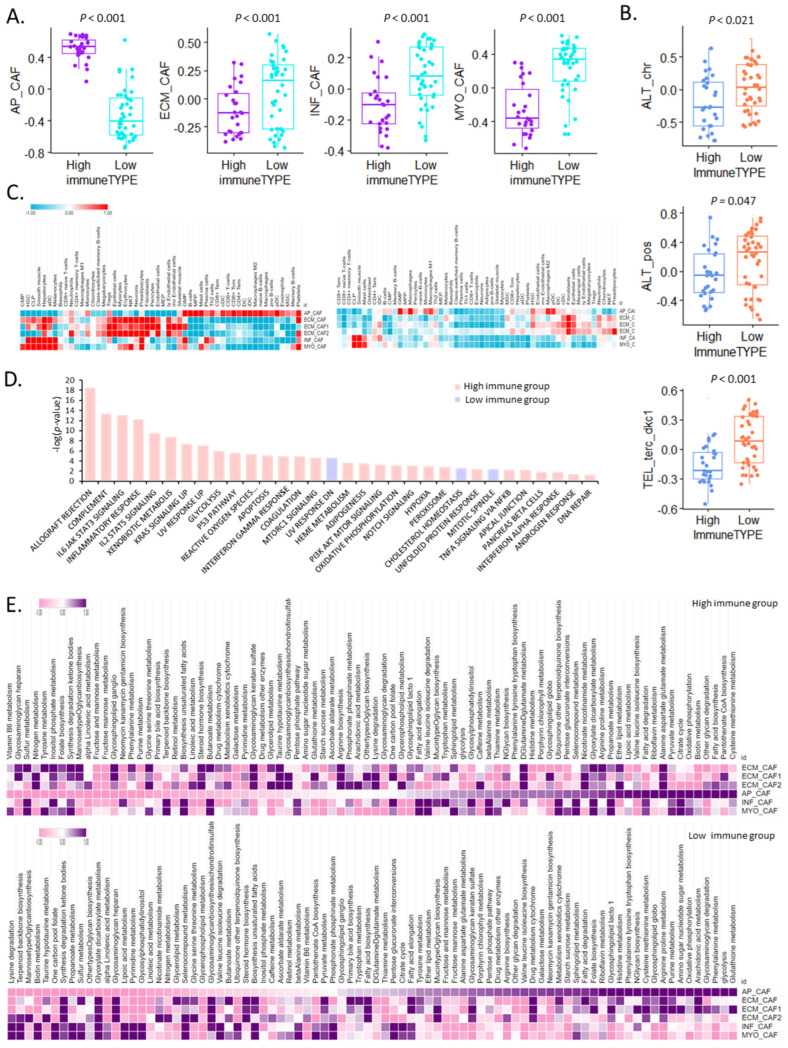
ApCAFs are enriched in the high-immunity group. (**A**) Box plot for apCAF, ecmCAF, infCAF, and myoCAF expression in the high- and low-immunity groups. (**B**) Box plot for telomere maintenance mechanism pathway activity in the high- and low-immunity groups. (**C**) Heat map of different CAF subtype cell types in the high- and low-immunity groups. (**D**) Bar graph of the *p*-value for cancer hallmarks in the two groups (pink, high-immunity group; sky blue, low-immunity group). (**E**) Heat map of 84 Kyoto Encyclopedia of Genes and Genomes metabolic pathways in the two groups (**top**, high-immunity group; **bottom**, low-immunity group).

Using bulk RNA-seq, we identified high- and low-immunity groups and examined the TIME and cancer hallmarks in each group. Notably, apCAFs were significantly more enriched in the high-immunity group than in the low-immunity group (*p* < 0.001), whereas ECM CAFs, infCAFs, and myoCAFs were significantly enriched in the low-immunity group (Figure 2A).

Moreover, the activity of telomere maintenance mechanism pathways was higher in the low-immunity group (Figure 2B). The correlation of CAFs with immune cells was examined, which revealed differences between the high- and low-immunity groups. In the high-immunity group, apCAFs were positively correlated with immune cells related to innate immunity (B, CD8 + T, CD4 + T, dendritic, naïve B, and memory B cells). The three ECM CAFs exhibited similar correlations with the immune cells. Regulatory T, epithelial, natural killer T, endothelial cells, and infCAFs were positively correlated with hematopoietic stem cells, smooth muscle cells, granulocyte/monocyte progenitor cells, and plasma cells. MyoCAFs positively correlated with common myeloid progenitor cells, mast cells, and platelets. The correlations with the low- and high-immunity groups differed. Cells associated with innate immunity negatively correlated with apCAFs and positively correlated with macrophages. CAF subtypes demonstrated distinct correlations with immune cells (Figure 2C).

We further investigated the mechanisms by which the high-immunity group was related to tumorigenesis in terms of cancer hallmarks and found that 32 of the 50 hallmark characteristics differed significantly (false discovery rate < 0.001) between the two groups. Three pathways (ultraviolet response downregulation, cholesterol homeostasis, and mitotic spindles) were significantly upregulated in the low-immunity group, whereas the remaining twenty-nine pathways were significantly upregulated in the high-immunity group (Figure 2D). These findings confirm that cancer hallmarks are more recognizable in the high-immunity group than in the low-immunity group.

Finally, we examined the association between CAF subtypes and metabolic reprogramming based on their immune status. apCAFs in the high-immunity group were associated with cysteine and methionine metabolism, pantothenate coenzyme A biosynthesis, and fatty acid biosynthesis, whereas those in the low-immunity group positively correlated with various metabolic pathways such as glutathione metabolism, glycolysis, and phenylalanine metabolism (Figure 2E). These findings imply that TIMEs contribute to energy metabolism via metabolic reprogramming through interactions with different CAF subtypes.

### 3.3. CAF Subtype-Specific Metabolic Reprogramming Results in Patient-Specific Phenotypes

**Figure 3:**
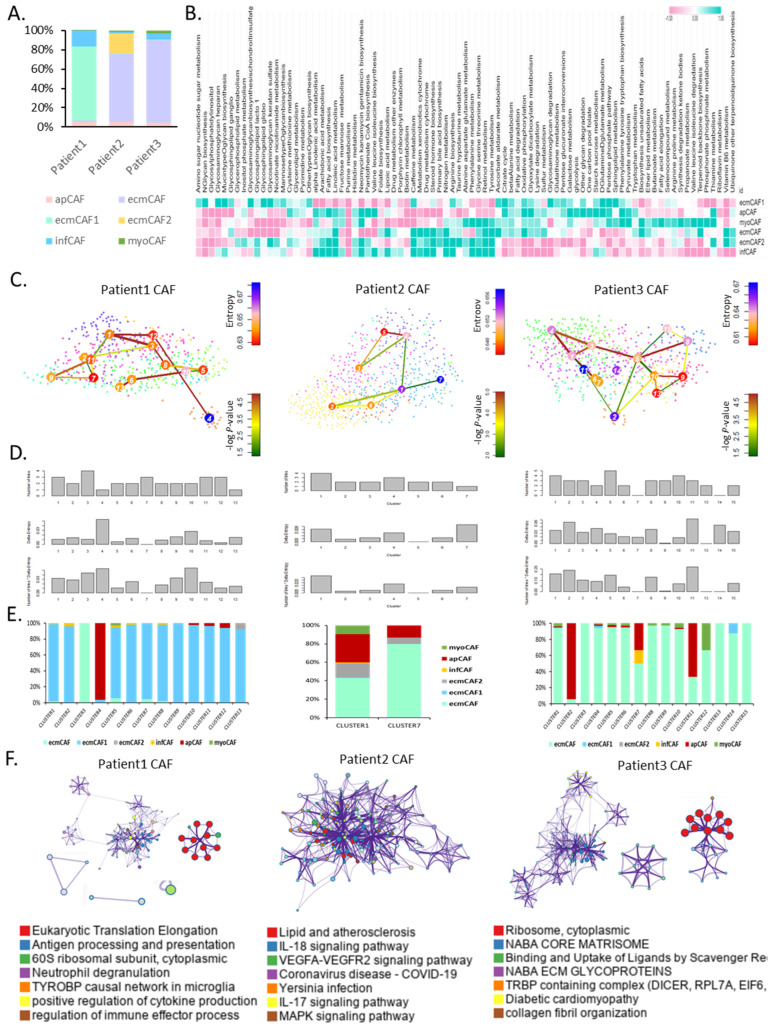
CAF subtypes show metabolic heterogeneity and distinct stem-like CAF signatures at the single-cell level. (**A**) Bar graph of a fraction of CAF subtypes at the single-cell level. (**B**) Heat map of metabolic reprogramming for CAF subtypes at the single-cell level. (**C**) tSNE plot for stem-like CAFs (**left**, patient 1; **middle**, patient 2; **right**, patient 3). (**D**) Bar graph of stemness in clusters (**top**, number of links; **middle**, Delta-Entropy; **bottom**, number of links + Delta-Entropy). (**E**) Bar graph of a fraction of CAF subtypes. (**F**) Gene ontology analysis network for highly differentially expressed genes (**left**, patient 1; **middle**, patient 2; **right**, patient 3). tSNE, t-distributed stochastic neighbor embedding.

We performed a single-cell analysis of metabolic reprogramming by CAF subtypes using 2373 CAFs from three patients with VS. Fractions of various CAF types were present in these patients. Although the percentages of apCAFs were comparable across all three patients, the proportions of heterogeneous ECM CAFs differed significantly between the three patients. All three patients had ECM CAF proportions > 50% of the total CAF (Figure 3A). Analysis of single cells facilitated a more specific analysis than that of bulk samples, which was of interest because different CAF types use different proportions of metabolic energy.

Specific pathways were enriched in apCAFs including the biosynthesis of neomycin, kanamycin, and gentamicin; valine, leucine, and isoleucine; and pantothenate coenzyme A. Metabolic pathways enriched in ecmCAF1s included glycosaminoglycan keratan sulfate, glycan biosynthesis, glycosaminoglycan degradation, and glycosaminoglycan heparan. Metabolic pathways enriched in ecmCAFs included steroid hormone biosynthesis, linoleic acid and caffeine metabolism, vitamin B6 and nitrogen metabolism, and linoleic acid and caffeine metabolism. Metabolic pathways enriched in myoCAFs included terpenoid backbone biosynthesis, thiamine metabolism, retinol metabolism, and phenylalanine, tyrosine, and tryptophan biosynthesis. The InfCAFs involved in nitrogen, caffeine, and linoleic acid metabolism exhibited patterns similar to those of ecmCAF2 (Figure 3B).

We determined the differentially expressed genes associated with stemness and calculated the transcriptomic entropy of CAFs for each patient using the StemID tool (Figure 3C). The fraction of apCAFs was relatively high in the extremely high stemness cluster (Figure 3D,E).

In patient 1, antigen processing and presentation, neutrophil degranulation, positive regulation of cytokine production, and regulation were all affected. The expression levels of genes involved in immune effector processes were relatively high. In patient 2, genes involved in lipid metabolism, atherosclerosis, the interleukin (IL)-18 signaling pathway, and the VEGFA-VEGFR2, IL-17, and MAPK signaling pathways were all expressed at relatively high levels. In patient 3, the expression levels of genes associated with the naba core matrix, naba ECM glycoproteins, and collagen fibril organization were relatively high (Figure 3F).

These findings present a therapeutic strategy that can determine CAF heterogeneity and the CAF subtype in each patient and potentially provide a cornerstone for the development of more specific treatment options for patients with VS from the perspective of precision medicine.

### 3.4. CAF Subtype Signature of VS Is Unique

**Figure 4:**
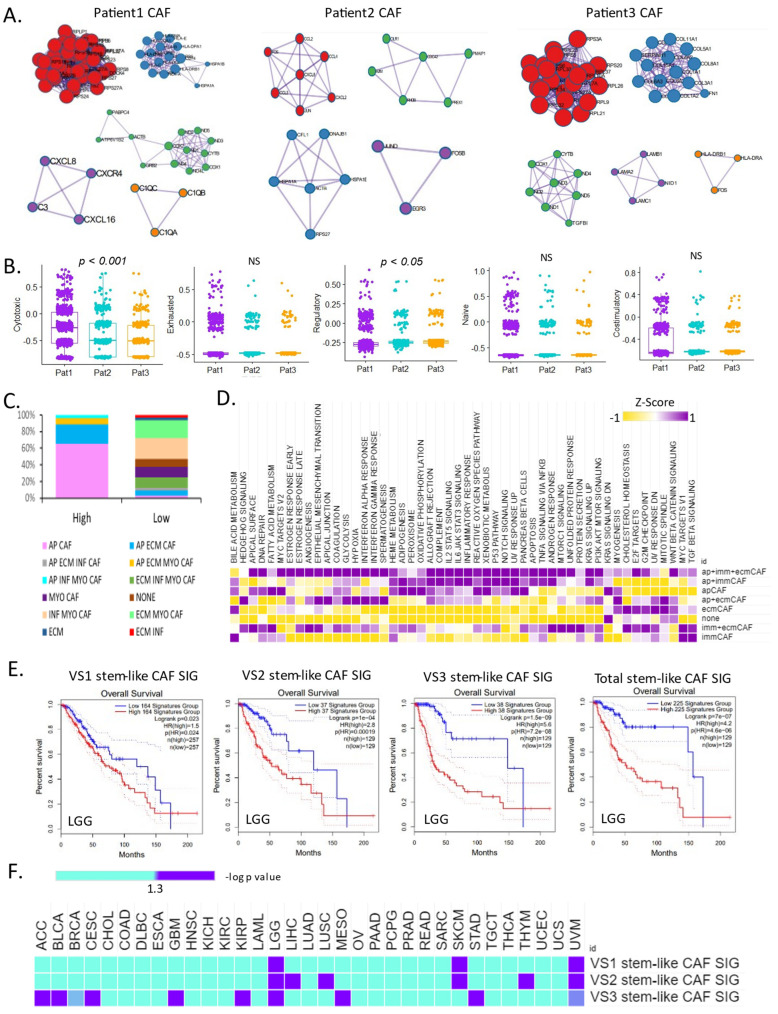
CAF subtype signatures predict patient prognosis in pan-cancer. (**A**) PPI network for stem-like CAF (**left**, patient 1; **middle**, patient 2; **right**, patient 3). (**B**) Box plot of T cell cytotoxicity, exhaustion, regulatory cytokines, naïve, and costimulation in three patients at the single-cell level. (**C**) Box plot of a fraction of CAF subtypes in the high- and low-immunity groups. (**D**) Heat map of cancer hallmark pathway activity in eight CAF subtypes in bulk samples. (**E**) Kaplan–Meier plots showing the overall survival rates for the high and low CAF subtype signatures in low-grade glioma. (**F**) Heat map of overall survival rate for the three CAF subtype signatures in The Cancer Genome Atlas Pan-Cancer.

We used the MCODE algorithm to perform PPI analysis to determine the CAF phenotype of each patient. Patient 1 was classified into the following five categories: classical transcription, elongation of eukaryotic translation, antigen processing and presentation, oxidative phosphorylation, and G-alpha signaling events. This CAF phenotype is associated with antibody-mediated activation of the complement system. Patient 2 was classified into four categories related to the IL-18 signaling pathway: negative regulation of inclusion body assembly, leukocyte differentiation, and signaling by NTRK1. Patient 3 was classified into five categories: positive regulation of integrin-mediated signaling pathways, Th1 and Th2 cell differentiation, cytoplasmic ribosomal proteins, collagen biosynthesis and modifying enzymes, and mitochondrial ATP synthesis coupled with electron transport (Figure 4A).

At the single-cell level, the features of T cell costimulation, regulatory cytokines and receptors, T cell exhaustion, and cytotoxicity in the three patients revealed that patient 1 had a greater proportion of cytotoxic T cells (Figure 4B). CAF subtypes at the single-cell level in patients with VS are valuable for identifying CAF characteristics in each patient using stem-like CAFs. We used the CAF gene signature identified at the single-cell level to classify patients from the bulk gene expression profiles of patients with VS and identified eight possible subtype classifications. The proportion of enriched apCAFs was highest in the high-immunity group, while that of enriched infCAFs and myoCAFs that were enriched was highest in the low-immunity group (Figure 4C).

We also examined bulk gene expression profiles in samples lacking CAF gene signature expression. The degree of enrichment of the cancer hallmark pathway in each patient group was determined via patient classification. We found that the hallmark cancer pathway was most enriched in the patient group with CAF characteristics, in which apCAF, immune CAF (immCAF), and ecmCAF signatures were expressed. The classification was similar in the groups wherein apCAFs, immCAFs, and ecmCAFs were all expressed, only apCAFs and immCAFs were expressed, and only apCAFs were expressed.

Although heterogeneity exists in cancer hallmark pathways, some pathways are commonly highly expressed (such as reactive oxygen species, xenobiotic metabolism, the p53 pathway, Notch signaling, and pancreatic beta cells). When only ecmCAFs were expressed, the cancer hallmark pathway was similar to that in the group lacking CAF signatures (Figure 4D). Our results were similar to those in which we confirmed the significantly higher apCAF levels in the high-immunity group compared with the low-immunity group (*p* < 0.001). The hallmark characteristics of cancer revealed a significantly higher difference; nonetheless, the CAF subtypes were distinguishable. Furthermore, we predicted the prognosis of low-grade gliomas using the gene signature identified in the CAF subtype of each of the three patients using single-cell data. These results differentiated the poor prognosis group from the high gene expression group (*p* = 0.023, VS1 stem-like CAF SIG; *p* = 1 × 10^−4^, VS2 stem-like CAF SIG; *p* = 1.5 × 10^−9^, VS3 stem-like CAF SIG; *p* = 7 × 10^−7^, VS total stem-like CAF SIG; Figure 4E). We used TCGA Pan-Cancer data to confirm that the CAF signatures (Supplementary Tables S1–S3) identified at the single-cell level aided prognosis. ApCAF and immCAF signatures were significantly (*p* < 0.001) more prognostic than ecmCAF signatures. In contrast, ecmCAF signatures in eight cancers confirmed poor prognosis in the high-expression group. We used the stem-like CAF signature to confirm prognosis in TCGA Pan-Cancer. VS3 stem-like CAF showed the largest differences (Figure 4F).

These results suggest that patients with VS can be classified from a precision medicine perspective using the CAF signature identified at the single-cell level. In addition, as a biomarker for predicting the TIME, the CAF signature can help predict tumor growth and development, thereby facilitating the diagnosis of patients.

## 4. Discussion

Solid stromal tumors that are not infiltrated by immune cells exhibit limited therapeutic responses to immunotherapy [17]. Several studies have examined the link between CAFs and the response to immune checkpoint inhibitors [18], as well as the prognosis of solid tumors [19,20]. A recent pan-cancer analysis of 226 samples from 10 types of solid cancers, which investigated the TME at single-cell resolution, revealed both the similarities and plasticity of heterogeneous CAFs [21]. However, studies on the classification and characterization of CAF subtypes in VS remain scarce. Therefore, we explored these aspects and identified marker genes that classified CAFs into six distinct subtypes. In addition, we confirmed the role of CAFs in antigen presentation during immune activation. Furthermore, the high activity of CAFs in glycolysis, the citrate cycle, and fatty acid biosynthesis suggests their involvement in the metabolic reprogramming of VS. Notably, bulk sample data analysis showed distinct upregulation (60%) of cancer hallmarks in the high-immunity group. CAFs displayed stem-like subtype characteristics in each patient, undergoing transitions with high entropy and stemness at the single-cell level. We classified patients based on the identified gene signature pattern at the single-cell level using stem-like CAFs. Additionally, cancer hallmarks were highly active in patient samples with high signatures for all three CAF subtypes (apCAF, immCAF, and ecmCAF). Finally, we found that the upregulation of the IL-6–IL-6R signaling pathway is a defining feature of infCAFs and is shared across multiple tumor types. We hypothesized that anti-IL-6 therapies, such as siltuximab and tocilizumab, may target these CAFs [22].

In conclusion, we employed a systematic approach to characterize various CAF subtypes and identify potential sources of CAFs. Additionally, we highlighted the various states of CAFs in relation to metabolic reprogramming and prognosis. The identified gene signature could inform the design of novel treatments targeting VS. CAFs may advance personalized treatment in precision medicine.

However, further experiments are needed to elucidate the precise role of each subtype and to trace its origin. Nonetheless, this is the first study to provide novel insights into the role of CAFs in VS and may lay the groundwork for the development of CAF-targeted therapies in the future.

## Data Availability

All data analyzed in this study are included in this published article (Xu, M.; Wang, S.; Jiang, Y.; Wang, J.; Xiong, Y.; Dong, W.; Yao, Q.; Xing, Y.; Liu, F.; Chen, Z.; et al. Single-cell RNA-Seq reveals heterogeneity of cell communications between Schwann cells and fibroblasts within vestibular schwannoma microenvironment. *Am. J. Pathol.* **2022**, *192*, 1230–1249. https://doi.org/10.1016/j.ajpath.2022.06.006).

## Supplementary Materials

The following supporting information can be downloaded at: https://www.mdpi.com/article/10.3390/cells13191669/s1, Figure S1: ApCAFs are primarily regulated by the transcription factors RFXANK, RFXAP, and RFX5 (Figure S1A). MyoCAFs are primarily regulated by POU5F1, TCF4M, and TFAP2A (Figure S1B); Table S1: Stem like CAF signature of patient1; Table S2: Stem like CAF signature of patient2; Table S3: Stem like CAF signature of patient3**.**

## References

[1] Liu L., Liu L., Yao H.H., Zhu Z.Q., Ning Z.L., Huang Q. (2016). Stromal myofibroblasts are associated with poor prognosis in solid cancers: A meta-analysis of published studies. PLoS ONE.

[2] Lockwood D.S., Yeadon T.M., Clouston A.D., Crawford D.G., Fawcett J., Callaghan S.A., Gotley D.C. (2003). Tumor progression in hepatocellular carcinoma: Relationship with tumor stroma and parenchymal disease. J. Gastroenterol. Hepatol..

[3] Kretzschmar K., Weber C., Driskell R.R., Calonje E., Watt F.M. (2016). Compartmentalized epidermal activation of beta-catenin differentially affects lineage reprogramming and underlies tumor heterogeneity. Cell Rep..

[4] Lavie D., Ben-Shmuel A., Erez N., Scherz-Shouval R. (2022). Cancer-associated fibroblasts in the single-cell era. Nat. Cancer.

[5] Davidson S., Efremova M., Riedel A., Mahata B., Pramanik J., Huuhtanen J., Kar G., Vento-Tormo R., Hagai T., Chen X. (2020). Single-cell RNA sequencing reveals a dynamic stromal niche that supports tumor growth. Cell Rep..

[6] Darby I.A., Zakuan N., Billet F., Desmoulière A. (2016). The myofibroblast, a key cell in normal and pathological tissue repair. Cell Mol. Life Sci..

[7] Guerrero-Juarez C.F., Dedhia P.H., Jin S., Ruiz-Vega R., Ma D., Liu Y., Yamaga K., Shestova O., Gay D.L., Yang Z. (2019). Single-cell analysis reveals fibroblast heterogeneity and myeloid-derived adipocyte progenitors in murine skin wounds. Nat. Commun..

[8] Chen P.Y., Wei W.F., Wu H.Z., Fan L.S., Wang W. (2021). Cancer-associated fibroblast heterogeneity: A factor that cannot be ignored in immune microenvironment remodeling. Front. Immunol..

[9] Sung J.Y., Cheong J.H. (2022). New immunometabolic strategy based on cell type-specific metabolic reprogramming in the tumor immune microenvironment. Cells.

[10] Xu M., Wang S., Jiang Y., Wang J., Xiong Y., Dong W., Yao Q., Xing Y., Liu F., Chen Z. (2022). Single-cell RNA-Seq reveals heterogeneity of cell communications between Schwann cells and fibroblasts within vestibular schwannoma microenvironment. Am. J. Pathol..

[11] Hao Y., Hao S., Andersen-Nissen E., Mauck W.M., Zheng S., Butler A., Lee M.J., Wilk A.J., Darby C., Zager M. (2021). Integrated analysis of multimodal single-cell data. Cell.

[12] Zhou Y., Zhou B., Pache L., Chang M., Khodabakhshi A.H., Tanaseichuk O., Benner C., Chanda S.K. (2019). Metascape provides a biologist-oriented resource for the analysis of systems-level datasets. Nat. Commun..

[13] Hänzelmann S., Castelo R., Guinney J. (2013). GSVA: Gene set variation analysis for microarray and RNA-seq data. BMC Bioinform..

[14] Grün D., Muraro M.J., Boisset J.C., Wiebrands K., Lyubimova A., Dharmadhikari G., van den Born M., van Es J., Jansen E., Clevers H. (2016). De novo prediction of stem cell identity using single-cell transcriptome data. Cell Stem Cell.

[15] Aran D., Hu Z., Butte A.J. (2017). xCell: Digitally portraying the tissue cellular heterogeneity landscape. Genome Biol..

[16] Tang Z., Kang B., Li C., Chen T., Zhang Z. (2019). GEPIA2: An enhanced web server for large-scale expression profiling and interactive analysis. Nucleic Acids Res..

[17] Gorchs L., Kaipe H. (2021). Interactions between cancer-associated fibroblasts and T cells in the pancreatic tumor microenvironment and the role of chemokines. Cancers.

[18] Sung J.Y., Cheong J.H. (2022). Intercellular communications and metabolic reprogramming as new predictive markers for immunotherapy responses in gastric cancer. Cancer Commun..

[19] Sung J.Y., Cheong J.H. (2022). The matrisome is associated with metabolic reprograming in stem-like phenotypes of gastric cancer. Cancers.

[20] Sung J.Y., Cheong J.H. (2022). Prognosis-related gene signature is enriched in cancer-associated fibroblasts in the stem-like subtype of gastric cancer. Clin. Transl. Med..

[21] Luo H., Xia X., Huang L.B., An H., Cao M., Kim G.D., Chen H.N., Zhang W.H., Shu Y., Kong X. (2022). Pan-cancer single-cell analysis reveals the heterogeneity and plasticity of cancer-associated fibroblasts in the tumor microenvironment. Nat. Commun..

[22] Karakasheva T.A., Lin E.W., Tang Q., Qiao E., Waldron T.J., Soni M., Klein-Szanto A.J., Sahu V., Basu D., Ohashi S. (2018). IL-6 mediates cross-talk between tumor cells and activated fibroblasts in the tumor microenvironment. Cancer Res..

