# Supplementary figures and images for: Cancer-Associated Fibroblast Subtypes Reveal Distinct Gene Signatures in the Tumor Immune Microenvironment of Vestibular Schwannoma
Source: Cells. 2024 Oct 9;13(19):1669. doi: 10.3390/cells13191669 (PMC11475780; doi:10.3390/cells13191669)

## Slide 1
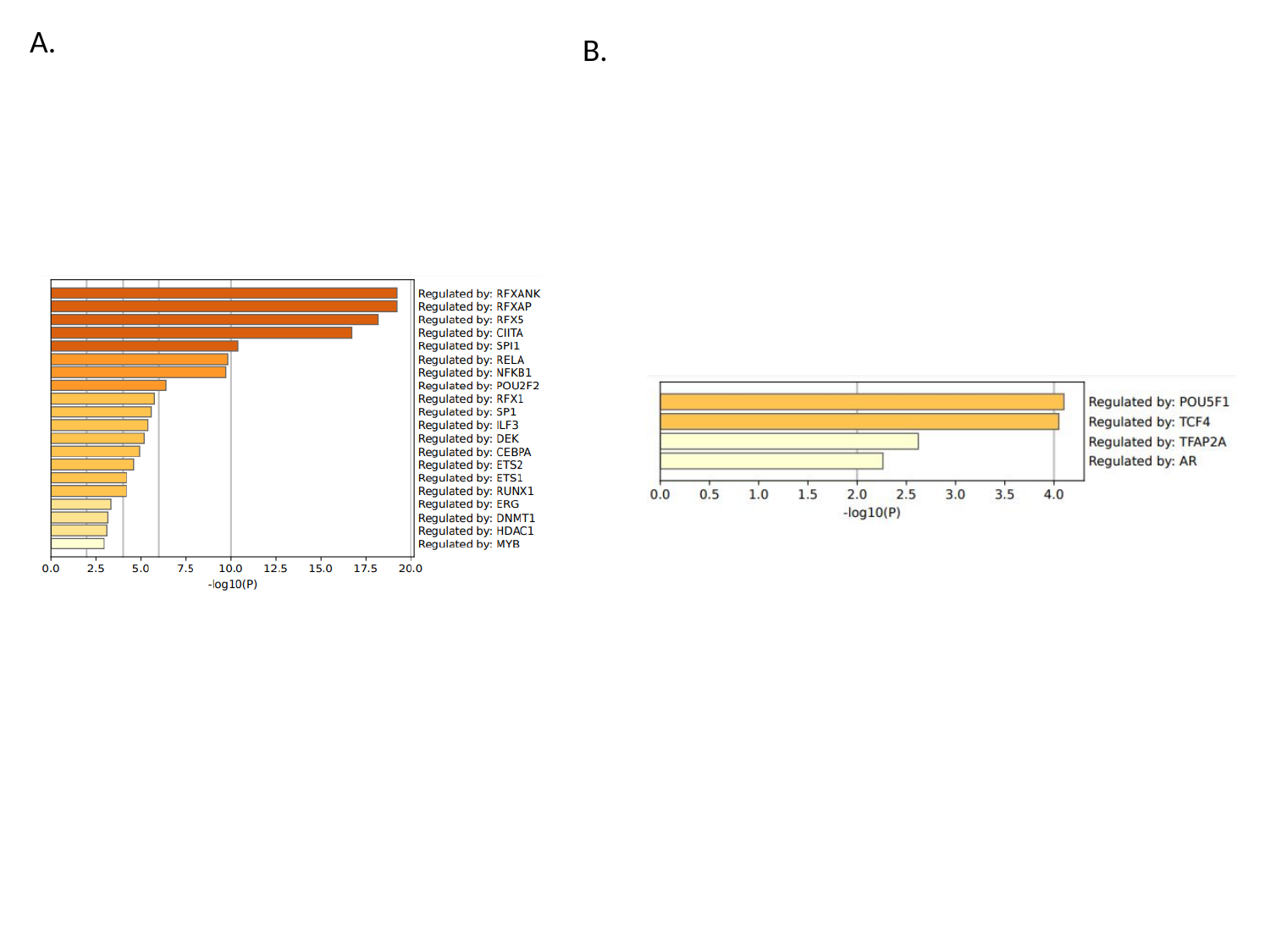

A.
B.

Supplement: Supplementary file 1 [file cells-13-01669-s001.zip › Supplementary Figure1.pptx]
